# Diagnostic accuracy of three placement sites for the cold test in subjects amongst different age groups

**DOI:** 10.1186/s12903-019-0878-2

**Published:** 2019-08-19

**Authors:** Brenda Eréndida Castillo-Silva, Jorge Alejandro Alegría-Torres, Gabriel Alejandro Martínez-Castañón, Carlo Eduardo Medina-Solís, Norma Verónica Zavala-Alonso, Nereyda Niño-Martínez, Eva Concepción Aguirre-López, Nuria Patiño-Marín

**Affiliations:** 1 Clinical Research Laboratory, Program of Doctorate in Dental Sciences, Faculty of Dentistry, San Luis Potosí University, Manuel Nava # 2. Zona Universitaria, PC 78290 San Luis Potosí, Mexico; 2 Department of Pharmacy, University of Guanajuato, Guanajuato, Mexico; 3 Program of Doctorate in Dental Sciences, Faculty of Dentistry, San Luis Potosí University, San Luis Potosí, Mexico; 4 Area of Dentistry of the Institute of Health’s Sciences, Autonomous University of The State of Hidalgo, Pachuca, Hidalgo Mexico

**Keywords:** Site, Cold test, Accuracy, Gender, Age

## Abstract

**Background:**

The cold test is a specific test of pulp sensitivity and is part of the endodontic diagnosis. The aim of this study was to identify the diagnostic accuracy including sensitivity, specificity, accuracy, positive predictive value and negative predictive value in three sites for the cold test in teeth with a need for endodontic treatment within different age groups from both genders.

**Methods:**

A cross-sectional study was performed, evaluating 425 subjects. Two hundred and fifty-eight subjects from both genders from the ages of 17–27, 28–39, 40–50, and 51–65 years-old participated in the study. The cold test studied was 1, 1, 1, 2-tetrafluoroethane, and the gold standard was established through direct pulp inspection. The sites evaluated in the study were: The sites evaluated in the study were: a) the middle third of the buccal surface; b) the cervical third of the buccal surface, and c) the middle third of the lingual surface.

**Results:**

The highest diagnosted accuracy was observed on the middle third buccal surface with an accuracy of = 0.97, a sensitivity of = 1.00, a specificity of 0.95, a predictive value of = 0.95 and a negative predictive value of = 1.00. This was in the female group aged from 40 to 50 years old.

**Conclusion:**

The tables of this study can be used as an auxiliary for pulp sensitivity tests.

## Background

The diagnosis of dental pulp status is necessary to decide endodontic treatment. The diagnosis should be considered as a synthesis of medical and dental history, clinical examination, specific tests, and a radiographic analysis [1]. The cold test is a specific test of pulp sensitivity and is part of the endodontic diagnosis [2]. The cold test evaluates the nervous response of the pulp and does not evaluate the pulp vascularity that is the indicator of pulp vitality. Therefore, a necrotic tooth can respond positively to the cold test with the presence of vital nerve fibers that remain in root canals, thus generating an incorrect diagnosis in the patient [2, 3]. To identify a correct diagnosis of a vital or necrotic pulp using the cold test, it is necessary to compare the results of the cold test with a gold standard (available and objective method) [2–6] and calculate: True-positives, true-negatives, false-positives, false-negatives [7–9], accuracy, sensitivity, specificity and predictive values [10–12]. a) The true positives are the number of cases correctly identified with necrotic pulps using the gold standard and cold tests; b) The true-negatives are the number of cases correctly identified with vital pulps with the gold standard and cold tests [7–10, 13]. The false results are incorrect diagnoses. c) The false-positives are the number of teeth with vital pulp diagnosed using the gold standard that were incorrectly diagnosed with necrotic pulp using the cold test; d) The false-negatives are the number of teeth with necrotic pulp diagnosed with the gold standard that were incorrectly diagnosed with vital pulp within the cold test [7–9]. The predictive values are calculated with the results that are true and false. e) The positive predictive value (PPV) is the probability that a tooth without a sensitive response represents a tooth with necrotic pulp within the cold test; and f) the negative predictive value (NPV) is the probability that a tooth with a sensitive response represents a tooth with vital pulp within the cold test [7–9, 13]. The predictive values are different from the sensitivity and specificity, because both predictive values are focused on the response of the cold test and not within the gold standard. The sensitivity indicates the ability of the gold standard to identify teeth with necrotic pulp and the specificity indicates the ability of the gold standard to identify teeth with vital pulp [7, 8, 10, 13]. On the other hand, the calculation of accuracy is necessary in the diagnostic tests. The accuracy is the correct result obtained from the cold test compared with the gold standard [10–12]. The concepts of accuracy and reproducibility are related but different. The reproducibility refers to the ability of the cold test to reproduce the same result in two different sites of the tooth. For example, a test could be reproducible but not accurate if on two occasions it produces roughly the same results, but those results could differ greatly from the actual value determined by the gold standard [11]. Authors identified false results or incorrect results in the diagnosis of teeth with vital and necrotic pulp in subjects with a need for endodontic treatment using pulp sensitivity tests [2, 5, 7, 14–20]. Some variables related with false results were: the placement sites for tests, the presence of diseases in the teeth, the gender and age [2, 7, 15, 16, 18]. It is necessary to perform studies that provide information to decrease the frequency of subjective responses or incorrect diagnostics using the cold test. We have not found publications in the last 20 years that report results related to the diagnostic accuracy of different sites for the cold test considering the gender and age of the subject. Therefore, the aim of this study was to identify the diagnostic accuracy including sensitivity, specificity, accuracy, positive predictive value and negative predictive value of 3 placement sites for the cold test in teeth with a need for endodontic treatment in subjects of both genders within different age groups.

## Methods

A cross-sectional study was performed from February 2015 to January 2017 in patients referred for endodontic evaluation at the Clinic of Dentistry at the San Luis Potosi University, San Luis Potosí, SLP, Mexico (located in Central Mexico). Following the Helsinki’s ethical principles declaration, an informed and voluntary written consent was obtained from the patients prior to the start of the study. The study was approved by the Research Ethics Committee of the Faculty of Dentistry, San Luis Potosi University, San Luis Potosi, SLP, Mexico. The participants were selected using a non-probability consecutive sampling. Four hundred and twenty-five subjects with no previous clinical diagnoses of pulp status were evaluated in the study. A total of 258 subjects fulfilled the following criteria: a) Inclusion. 1) Subjects without diagnosed systemic diseases; 2) both genders; 3) 17–65 years old; 4) without the use of drugs (narcotics, alcohol or nonsteroidal anti-inflammatory drugs) for 24 h prior to the study; 5) subjects with teeth with a need for endodontic treatment (vital and necrotic pulp). b) Exclusion. 1) patients with a recent history of orthodontic treatment or trauma in the last 6 months; 2) teeth with full surface crowns; 3) restorations in the surfaces under evaluation; 4) cavity access; 5) calcified root canals; 7) incomplete root formation [5–7]. The subjects were divided into two groups: 1) a female group and 2) a male group. Both groups were subdivided into 4 age groups: 17–27, 28–39, 40–50, and 51–65 years forming a total of eight groups.

### Calculation of sample size

The sample size was calculated with a two-factor design (two-way ANOVA). One factor (age) consisted of eight experimental groups (17–27, 28–39, 40–50, and 51–65 years). It was determined that 20 subjects per group was the minimum sample size with a power of 0.8 and a significance level of 0.05 [18].

All participants were evaluated using the following protocol:

1. *Medical and dental history.* A researcher recorded the medical history (including details of any medication taken within the last 6 months), a dental history (past and recent treatments), took into account major discomforts (onset, duration, frequency, intensity and location of pain), performed a clinical examination (coronal evaluation for caries, restorations, enamel loss [abrasion, attrition or fractures]), performed a periodontal evaluation (periodontal probing, gingivitis, and abscesses), and performed a radiographic analysis (size and shape of the pulp chamber and root canals, radiolucent lesions, or thickened periodontal ligaments).
2. *Cold pulpal testing.* The teeth were isolated with cotton rolls, and dried with cotton gauze [21, 22]. A clinical researcher sprayed a no. 2 cotton pellet with a refrigerant spray (1, 1, 1, 2-tetrafluoroethane. Hygienic Endo-Ice® Green [Endo-Ice]; Coltene Whaledent, Cuyahoga Falls, OH) [6, 7, 19], which was then placed onto the crown of the tooth for 25 s or until the participant raised a hand to indicate a cold sensation. The previous procedure was performed on three sites of the tooth: 1) in the center of the middle third of the buccal surface (MTBS); 2) in the center of the cervical third of the buccal surface (CTBS), and 3) in the center of the middle third of the lingual surface (MTLS). To ensure pulp recovery after the first site, the researchers allowed 5 min between each site [7, 10]. The results of the test with vital pulp were reported in seconds. a) First time evaluation (FT): The number of seconds from the application of the stimulus until the participant raised a hand. b) Second time evaluation (ST): The number of seconds between the removal of the stimulus until the absence of the sensation [7].
3. *Procedure of the test application.* The cold test was performed by 2 researchers who were blind to the subjects’ clinical signs and symptoms, dental history, periodontal status, and radiographic findings. In the application procedure of the test, one of the researchers recorded the response of the test in seconds and was blind to the site of the test application, and a second researcher applied the test to different sites randomly [23] and was blind to the response in seconds of the test. Each participant was instructed to raise his/her hand as soon as he/she felt a sensation during testing and lower his/her hand at the moment of the absence of the sensation [7]. The patient was blind to the response in seconds from the different sites.
4. *Pulp diagnosis with the gold standard*. Pulp status was determined using the gold standard. After completing the tests, endodontic treatment was performed, with the patient anesthetized with 2% of lidocaine and 1: 100,000 of diluted epinephrine. The gold standard of vital pulp or necrotic pulp was detected by opening the pulp chamber, and the pulp status was recorded through the observation of bleeding within the pulp chamber. The presence of the pulp blood flow represented vital pulp and the absence of it, represented a necrotic pulp. When vital tissue was identified in the apice, but necrotic tissue was observed in the chamber, the tooth was diagnosed as necrotic [2, 5, 6, 24]. The subjects of the study were slated for endodontic treatment.

### Statistical analysis

We obtained the frequencies and percentages of qualitative variables. We calculated mean ± standard deviation for the continuous variables. The true-positive, true-negative, false-positive, and false-negative were identified. The sensitivity, specificity, predictive values, accuracy, and prevalence were calculated. The reproducibility between sites on the tooth was determined with the kappa simple test (Cohen). Correlations (Spearman rho) were calculated with response times of vital pulps (FT and ST) between the sites [7]. The analyses were performed with Stata statistical software (Version 11; StataCorp, College Station, TX).

## Results

Four hundred and twenty-five subjects were invited to participate in the study, and 258 were included because they met the selection criteria. The subjects were divided into 8 groups: 1) 38 female (27%) from 17 to 27 years (21 ± 3.4); 2) 37 female (26%) from 28 to 39 years (34 ± 3.2); 3) 34 female (24%) from 40 to 50 years (44 ± 3.7); 4) 31 female (23%) from 51 to 65 years (57 ± 4.7); 5) 33 male (28%) from 17 to 27 years (22 ± 3.5); 6) 36 male (30%) from 28 to 39 years (34 ± 4.1); 7) 29 male (25%) from 40 to 50 years (44 ± 3.8) and 8) 20 male (17%) from 51 to 65 years (61 ± 3.7). Table 1 shows the teeth with vital and necrotic pulp according to the gold standard and the clinical evaluation in the female and male group from 17 to 65 year olds. In each study group, 20 teeth with vital pulp and 20 with necrotic pulp were identified with the gold standard with a total of 40 teeth per group. Within the 8 groups, 320 teeth were evaluated; 160 teeth with vital pulp and 160 with necrotic pulp. The most frequent teeth included in the study were the posterior teeth (premolars and molars). Dental caries was the most common pathology identified in 179 teeth. The sensitivity, specificity, predictive values, accuracy, false-positives, and false-negatives in the three placement sites for the cold tests of the 8 groups are shown in Table 2. The overall diagnostic accuracy in the three placement sites was: 1. MTBS: sensitivity = 0.94, specificity = 0.75, accuracy = 0.85, PPV = 0.80, and NPV = 0.92; 2. CTBS: sensitivity = 0.91, specificity = 0.81, accuracy = 0.86, PPV = 0.84, and NPV = 0.91 and MTLS: sensitivity = 0.96, specificity = 0.72, accuracy = 0.81, PPV = 0.76, and NPV = 0.95. The sites with sensitivity, specificity and predictive values ≥0.70 were observed in all the subjects of both genders within different age groups. Only in the male group from 51 to 65 years, values were identified from 0.25 to 0.65 in the specificity and in the positive predictive values. The highest values of the accuracy variable were observed in the MTBS site of the female group from 40 to 50 years (0.97) and in the CTBS site of the male group from 17 to 27 years (0.90). The highest diagnostic accuracy including sensitivity (1.00), specificity (0.95), predictive values (PPV = 0.95 and NPV = 1.00) and accuracy (0.97) was identified in the MTBS site of the female group from 40 to 50 years. In relation to the false positives and negatives, the highest values of false positives were observed in the MTLS site in females from the 17–27 years group (frequency = 8), and in the MTLS site for males in the 51–65 years group (frequency = 15). The false negatives with the highest values were observed in the CTBS site of the females from 51 to 65 years (frequency = 4) and in the CTBS site of males from 17 to 27 years (frequency = 3). Table 3 shows the results in seconds of the cold test in the three sites of the teeth with a need of endodontic treatment diagnosed with the gold standard as vital pulp in the study groups. The longest response in seconds from the application of the stimulus until the participant raised a hand was 20 s in the middle third of the lingual surface in the groups of female subjects from 17 to 27 years. In terms of the second time, the longest response from removing the stimulus until the absence of sensation was 20 s in the middle third of the cervical surface and the middle third of the lingual surface in the group of female subjects from 28 to 39 years. Table 4 shows the reproducibility between the sites in the study groups and the correlation of the times between sites in teeth with vital pulp. The value of higher reproducibility between sites was 0.90 between the CTBS vs MTLS in the female group from 40 to 50 years and 0.85 between the MTBS vs CTBS in males from 17 to 27 years. The strongest correlation was observed in the second time of the MTBS vs CTBS sites in the male group from 28 to 39 years *(*rho = 0.72*, p < 0.05).*

**Table 1:** Teeth with vital and necrotic pulp (according to the gold standard) and the clinical evaluation in the female and male groups from 17 a 65 years old

Diagnosis: Ideal Standard	Frequency (%)									
	*Female groups* 140 (54)					*Male groups* 118 (46)				
	Vital Pulp *n* = 80		Necrotic Pulp *n* = 80			Vital Pulp *n* = 80		Necrotic Pulp *n* = 80		
Years	Anterior Teeth	Posterior Teeth	Anterior Teeth	Posterior Teeth	*Total*	Anterior Teeth	Posterior Teeth	Anterior Teeth	Posterior Teeth	*Total*
17–27^a^	0	20 (100)	0	20 (100)	40	0	20 (100)	8 (40)	12 (60)	40
28–39^a^	0	20 (100)	7 (35)	13 (65)	40	0	20 (100)	4 (20)	16 (80)	40
40–50^a^	4 (20)	16 (80)	5 (25)	15 (75)	40	5 (25)	15 (75)	5 (25)	15 (75)	40
51–65^a^	1 (5)	19 (95)	8 (40)	12 (60)	40	6 (30)	14 (70)	14 (70)	6 (30)	40
Total	5 (6)	75 (94)	20 (26)	60 (74)	160	11 (13)	69 (87)	31 (38)	49 (62)	160
Clinical Evaluation										
Healthy^b^	0 (0)	0 (0)	0 (0)	0 (0)	0	1 (1)	2 (3)	0 (0)	0 (0)	3
Caries	4 (5)	42 (53)	10 (13)	40 (50)	96	4 (5)	35 (44)	11 (13)	33 (42)	83
Caries and Restoration	1 (1)	13 (16)	0 (0)	8 (10)	22	0 (0)	18 (22)	1 (1)	6 (8)	25
Restoration	0 (0)	16 (20)	2 (3)	11 (13)	29	1 (1)	12 (15)	2 (3)	5 (6)	20
Enamel loss	0 (0)	4 (5)	8 (10)	1 (1)	13	5 (6)	2 (3)	17 (21)	5 (6)	29

^a^*n* = 20 teeth, ^b^apparently healthy crown

**Table 2:** Sensitivity, Specificity, Predictive values, Accuracy, False-positives, and False-negatives in the three placement sites for cold tests of the 8 study groups

Groups for age	Sensitivity	Specificity	PPV	NPV	Accuracy			Sensitivity	Specificity	PPV	NPV	Accuracy		
	*Groups Female*					FP	FN	*Groups Male*					FP	FN
17–27														
MTBS	1.00	0.85	0.87	1.00	0.93	3	0	0.90	0.80	0.82	0.89	0.85	4	2
CTBS	0.95	0.90	0.90	0.95	0.92	2	1	0.85	0.95	0.95	0.86	0.90	1	3
MTLS	1.00	0.75	0.72	1.00	0.80	8	0	0.90	0.75	0.70	0.86	0.75	8	2
28–39														
MTBS	0.90	0.80	0.82	0.89	0.85	4	2	1.00	0.75	0.80	1.00	0.88	5	0
CTBS	0.85	0.95	0.95	0.86	0.90	1	3	1.00	0.75	0.80	1.00	0.88	5	0
MTLS	0.95	0.85	0.86	0.95	0.90	3	1	0.95	0.75	0.79	0.94	0.85	5	1
40–50														
MTBS	1.00	0.95	0.95	1.00	0.97	1	0	0.95	0.75	0.79	0.94	0.85	5	1
CTBS	1.00	0.75	0.80	1.00	0.87	5	0	0.95	0.75	0.79	0.94	0.85	5	1
MTLS	1.00	0.75	0.74	1.00	0.83	7	0	1.00	0.75	0.80	1.00	0.87	5	0
51–65														
MTBS	0.85	0.85	0.85	0.85	0.85	3	3	0.95	0.30	0.57	0.85	0.62	14	1
CTBS	0.80	0.95	0.94	0.83	0.88	1	4	0.95	0.50	0.65	0.90	0.72	10	1
MTLS	0.90	0.95	0.95	0.91	0.92	1	2	1.00	0.25	0.57	1.00	0.62	15	0

*PPV* positive predictive values, *PPN* negative predictive values, *MTBS*, Site Middle third of the buccal surface, *CTBS* Site Cervical third of the buccal surface, *MTLS* Site Middle third of the lingual surface, *FP* Frequency of false positives, *FN* Frequency of False Negatives

**Table 3:** Results of the cold test in seconds in the three sites with vital pulp (gold standard) of the 8 groups divided by gender and age

Sites	Middle third of the buccal surface	Cervical third of the buccal surface	Middle third of the lingual surface	
	Mean ± SD (Rank)			Total
Female groups				
17–27^a^				
FT	2.8 ± 1.4 (1–10)	3.3 ± 2.3 (1–15)	6.8 ± 5.5 (2–20)	4.3 ± 3.0 (1–20)
ST	4.4 ± 1.5 (2–7)	5.0 ± 2.2 (1–10)	3.6 ± 2.2 (1–7)	4.3 ± 1.9 (1–10)
28–39 ^a^				
FT	2.6 ± 1.4 (1–15)	2.5 ± 1.3 (1–15)	3.1 ± 2.2 (1–8)	2.7 ± 1.6 (1–15)
ST	4.7 ± 3.3 (1–15)	5.0 ± 4.4 (1–20)	4.9 ± 3.8 (1–20)	4.8 ± 3.8 (1–20)
40–50 ^a^				
FT	4.2 ± 3.9 (1–18)	2.9 ± 1.4 (1–10)	4.1 ± 3.5 (1–12)	3.7 ± 2.9 (1–18)
ST	4.7 ± 3.9 (1–15)	6.1 ± 4.7 (1–16)	4.8 ± 3.6 (1–13)	4.8 ± 3.6 (1–13)
51–65 ^a^				
FT	3.2 ± 2.3 (1–11)	2.7 ± 1.6 (1–10)	4.5 ± 3.5 (1–13)	3.4 ± 2.4 (1–13)
ST	6.2 ± 3.2 (1–12)	7.3 ± 3.5 (2–15)	5.1 ± 3.5 (1–15)	6.2 ± 3.4 (1–15)
Male groups				
17–27 ^a^				
FT	3.2 ± 2.4 (1–11)	3.0 ± 2.2 (1–11)	4.2 ± 2.2 (1–10)	3.4 ± 2.2 (1–11)
ST	3.6 ± 2.0 (1–8)	4.3 ± 1.8 (1–8)	3.4 ± 2.3 (1–9)	3.7 ± 2.0 (1–9)
28–39 ^a^				
FT	2.7 ± 1.4 (1–6)	2.9 ± 1.3 (1–9)	3.4 ± 2.7 (1–10)	3.0 ± 1.8 (1–10)
ST	5.3 ± 2.3 (1–10)	5.5 ± 3.4 (1–14)	4.9 ± 2.7 (2–18)	5.2 ± 2.8 (1–18)
40–50 ^a^				
FT	2.5 ± 1.3 (1–10)	2.6 ± 1.6 (1–10)	2.5 ± 1.5 (1–11)	2.5 ± 1.4 (1–11)
ST	5.5 ± 2.7 (1–10)	6.0 ± 3.5 (1–12)	5.4 ± 2.9 (1–12)	5.6 ± 3.0 (1–12)
51–65 ^a^				
FT	3.8 ± 2.3 (1–8)	3.9 ± 1.7 (1–7)	4.2 ± 3.4 (1–10)	3.9 ± 2.4 (1–10)
ST	5.8 ± 4.0 (1–14)	3.6 ± 2.5 (2–9)	4.0 ± 2.5 (1–8)	4.4 ± 3.0 (1–14)
Total	4.07 ± 2.4 (1–18)	4.16 ± 2.4 (1–20)	7.06 ± 3.0 (1–20)	–

*SD* Standard Deviation, *FT* First time in seconds, *ST* Second time in seconds

^a^Years

**Table 4:** Reproducibility between the sites in the study groups and the correlation of the times between sites in teeth with vital pulp

Reproducibility^a^			
Comparison between sites	Middle third of the buccal surface vs Cervical third of the buccal surface	Middle third of the buccal surface vs Middle third of the lingual surface	Cervical third of the buccal surface vs Middle third of the lingual surface
Female Groups			
17–27^b^	0.80	0.73	0.64
28–39^b^	0.70	0.80	0.70
40–50^b^	0.80	0.70	0.90
51–65^b^	0.85	0.75	0.80
Male Groups			
17–27^b^	0.85	0.75	0.80
28–39^b^	0.70	0.74	0.74
40–50^b^	0.79	0.74	0.74
51–65^b^	0.43	0.61	0.25
Correlation of times		Rho FT (Rho ST)	
Female Groups			
17–27^b^	0.62 -----		
28–39^b^	0.48 (0.45)	----- (0.54)	----- (0.51)
40–50^b^	----- (0.71)		
51–65^b^	0.57 (0.71)	0.50 -----	0.61 -----
Male Groups			
28–39^b^	----- (0.72)		
40–50^b^	----- (0.70)		

Rho FT = Correlation of Spearman with *p < 0.05* of the first time in seconds. Rho ST = Correlation of Spearman with *p < 0.05* of the second time in seconds

^a^Test of *Kappa* (Cohen). ^b^Years

## Discussion

To identify the diagnostic accuracy of 3 sites for the cold test including sensitivity, specificity, accuracy, positive predictive value and negative predictive value; it is convenient to have a gold standard. The standard is an objective method to perform a diagnosis [4]. The results of the gold standard should be compared with other methods or new tests to confirm that the results of the other methods or new tests are objective [7, 9]. Authors have reported that the gold standard for determining the actual state of dental pulp is a histological examination. The implementation of this standard requires the extraction of the tooth, which is impossible in most cases where the elimination of the tooth is not indicated. The gold standard *in clinical dentistry,* when diagnosing a vital or necrotic pulp with a need for endodontic treatment is done through the direct inspection of the pulpal blood flow within the pulp chamber. The presence of bleeding indicates vital pulp and the absence indicates necrotic pulp. When vital tissue is identified at the apex, but necrotic tissue is observed in the chamber, the tooth is diagnosed as necrotic [2, 5, 6, 10]. The direct inspection of the pulpal blood flow in teeth with a need for endodontic treatment represents the gold standard from the cold test through the following: The microcirculation and nerve fibers are two components of pulpal inflammation. The microcirculation within the dental pulp initiates an inflammatory response as part of a complex defensive mechanism to maintain the integrity and health of the dental pulp. This inflammatory reaction induces vasodilatation and increased pulpal blood flow. On the other hand, any increase in the blood flow and vascular permeability can cause major changes in the pressure inside the pulp chamber, which may compress blood vessels and lead to a decrease in the pulp flow. In terms of the nerve fibers, within the pulp vascularized by the blood flow, nerve bundles diverge and branch out towards the pulpo-dentin border. Nerve divergence continues until each bundle loses its integrity, and smaller fiber groups travel towards the dentine. The nerve fibers transmit the nerve impulse causing a positive response of the test by the stimulus. Therefore, the inflammatory response including the pulp blood flow and nerve fibers within the pulp chamber represents a positive response with the cold test [4, 24]. On the other hand, the direct inspection of the pulpal blood flow is the gold standard of teeth with vital pulp with a need for treatments without specifying the state of pulp inflammation. This gold standard should not be considered for teeth with healthy vital pulp because the direct inspection of the pulp chamber is not justified [2, 7, 15, 16, 18].

In this study, the teeth needed endodontic treatment; thus, the direct inspection of the pulpal blood flow was observed and the prevalence of necrotic pulp was 50% [7, 8, 10, 13].

The highest diagnostic accuracy including sensitivity (1.00), specificity (0.95), predictive values (PPV = 0.95 and NPV = 1.00) and accuracy (0.97) was identified in the MTBS site of the female group from 40 to 50 years. The sites with sensitivity, specificity and predictive values ≥0.70 and accuracy ≥0.75 were observed in all groups. Only in the male group from 51 to 65 years values of 0.25 to 0.65 were identified in specificity and in positive predictive values. In the accuracy, values from 0.62 to 0.72 were identified. Several authors reported differences between male and female subjects [16, 18, 20]. The thicker dentin and larger diameters of the crown in males may increase the time of the response to a stimulus to reach the pulpal nerves, resulting in a higher threshold [18]. Other variables to consider are: psychological and behavioral factors (apprehension or Hawthorne effect) and biological factors (hormonal levels and neurological differences). On the other hand, researchers did not report differences between genders [16, 25, 26]. In terms of age, the sites with predictive values ≥0.70 and accuracy ≥0.75 were observed in all the age groups, but the highest values of false positives were identified in the male group from 51 to 65 years (frequency = 15) and the false negatives in the female group from 51 to 65 years (frequency = 4). Researchers have identified diagnostic accuracy values of the pulp sensibility tests in subjects with different ages. One study evaluated 656 patients from ages 6 to 85 years. The results of pulpal sensibility tests (MTBS site), the tooth number, age, sex, number of restored surfaces, presence or absence of clinical or radiographic caries, and reported recent use of analgesic medications, were recorded. Authors observed that patients from 21 to 50 years showed a more accurate response to cold testing (accuracy = 0.904; sensitivity = 0.916; specificity = 0.896; positive predictive value = 0.862; and negative predictive value = 0.937) [2]. Some authors reported that due to the aging process, a higher frequency of collagenous fibers in the pulp, fewer blood vessels, nerve fiber degeneration, an increase in dentin sclerosis, an increase in the width of the secondary dentin, and a higher incidence of coronal calcifications were observed [2, 16, 27–30]. When researching the literature, we did not find studies with populations of both genders including different age ranges. The results obtained from these studies could be a possible complement to facilitate the endodontic diagnosis in the clinic.

The false results of the tests are limitations in studies of diagnostic accuracy. The teeth with false responses in the present study was of 15.6%, (*n* = 960 sites). Each tooth had at least one of the following clinical characteristics: extensive caries, reduced pulp chamber, a periodontal pocket, enamel loss, restorations and asymptomatic teeth before the consultation. Some possible explanations of false results related to clinical characteristics are: 1) Caries. Teeth may fail to respond to a vitality test because of the insulating properties of dentin reparative [7, 31]. On the other hand, the increased responsiveness to cold is a consequence of inflammation from an immune response resulting in decreased action potential thresholds [2]. 2) Smaller pulp chambers. The thermal stimulation of the pulp might be difficult in teeth with smaller pulp chambers. Teeth with caries can produce a formation of tertiary dentin that can lead to a narrowing in the pulp chamber [32]. 3) The pocket and periodontal attachment loss could promote pulp recession and increase the formation of reactionary dentin acting as an insulator and on the other hand, an increase to the response of the test through the exposure of cementum and dentin [1, 14]. 4) Enamel loss. One possible hypothesis regarding abrasion is that teeth with partial necrosis and abrasion facilitate the response of the remaining vital pulp with the cold test. The exposed dentin is related to an increased sensitivity to a stimulus [23, 31]. 5) Necrotic teeth may have responded to tests because of the presence of deep restorations or remaining vital nerve fibers [2, 3]. 6) Multirooted teeth. The state of partial necrosis in any root is related to false results [2, 7, 12]. 7) Asymptomatic teeth. In an asymptomatic pulp, the odds of having lingering pain caused by cold stimuli are 0.6 times less than for a similar patient diagnosed with symptomatic pulpal [33].

The placement site for cold test is a variable related with false results. In the study three sites were evaluated MTBS, CTBS, and MTLS. The 3 sites were selected for the following reasons: Most of the studies placed the stimulus on the MTBS, because this site had shown to have the least electrical resistance. Also, several authors reported a high accuracy on this site [5, 7, 10, 14–17, 21, 31, 34–36]. However, some studies indicate that thermal tests are more accurate when placed on the CTBS because this location represents the thinnest aspect of the enamel and the closest distance to the pulp chamber [7, 19, 23]. The middle third of the lingual surface was evaluated using an electrical test by proximity to the pulp horns [16, 20, 26, 36]. The incisal edge or occlusal surface are sites that have been analyzed by different studies. Researchers have reported high and low values of sensitivity, specificity, accuracy, positive predictive value and negative predictive value with cold and electric tests [16, 22, 26]. On this study we are not evaluating the incisal edge or occlusal surface, since several authors have reported high frequencies of caries, restoration, enamel loss (abrasion) and exposed dentin on those sites [37, 38]. All these clinical pathologies are associated with false results when using pulp sensibility tests [7, 22]. Therefore, we decided not to include said sites on this study.

Reproducibility (Precision) could decrease the frequency of false results. Reproducibility is the identification of two equal results performed in two different sites [11]. With reproducibility, the clinician could confirm a diagnosis using a different site to reduce the possibility of false results. The site with more diagnostic accuracy in the study including sensitivity (1.00), specificity (0.95), predictive values (PPV = 0.95 and NPV = 1.00) and accuracy (0.97) was the MTBS site from the female group from 40 to 50 years and the site with the highest reproducibility of the MTBS was CTBS (Kappa = 0.80 Table 4). Therefore, the diagnosis with the cold test in the female group from 40 to 50 years should be performed in the MTBS site and the diagnosis of the MTBS should be confirmed in the CTBS site. Several authors reported high values of reproducibility in the MTBS site with the cold test [7, 19].

In relation to the present study, the following aspects were considered: a) A gold standard was included in the calculations to obtain objective results related to the diagnosis of a vital or necrotic pulp in teeth with a need for treatment; b) The basic concepts were defined for the interpretation of results; c) The calculations for sensitivity, specificity, predictive values, accuracy, and reproducibility were performed according to the guidelines established in the literature; d) Three sites were included in the study; e) The measurement of the variables was conducted by 2 researchers to get independent results; f) The study was blind, and the application of the test at different sites was random; g) The researchers allowed at least 5 min to elapse after each pulp test so that the pulp could recover; h) The stimulus was placed in the tooth for 25 s or until the participant raised a hand [2, 5–7, 10, 15, 21, 23, 34] and i) Eight groups were formed to identify the response of the test in subjects of both genders with different age ranges.

The results reported in the tables of this study are a reference for health professionals (clinical application). For example, the site with an accurate diagnosis in the study for cold tests in a male subject between the ages of 40 and 50 years is the MTLS (Table 2. Accuracy = 0.87, PPV = 0.80, NPV = 1.00, Sensitivity = 1.00 and Specificity = 0.75). The highest values of diagnostic accuracy allow the identification of the most appropriate site. The site that confirms the diagnosis of MTLS is MTBS or CTBS (Table 4. Reproducibility of MTBS = 0.74 and CTBS = 0.74, both with the same value). The highest values of reproducibility allow the identification of a site to allow a confirmation of a diagnosis. With the subject’s gender and age, the clinician can identify the most appropriate site (the site with the highest diagnostic accuracy) to perform the test and the site to confirm the diagnosis (tables of results). Therefore, the tables of this study can be used as an auxiliary for pulp sensitivity tests, and tests can be used to complement a pulp diagnosis.

## Conclusions

a) Overall, the sites with sensitivity, specificity and predictive values ≥0.70 and accuracy ≥0.75 were observed in all groups. Only in the male group from 51 to 65 years, values from 0.25 to 0.65 were identified in specificity and in positive predictive values. As for the accuracy, values from 0.62 to 0.72 were observed. b) The highest diagnostic accuracy including sensitivity (1.00), specificity (0.95), predictive values (PPV = 0.95 and NPV = 1.00) and accuracy (0.97) was identified in the MTBS site of the female group from 40 to 50 years. c) The longest responses in seconds from the application of the stimulus until the participant raised a hand and that of the elimination of the stimulus until the absence of sensation were of 20 s. d) To confirm the response of the MTBS in the female group from 40 to 50 years, the best site was CTBS.

## Data Availability

The datasets used and/or analysed during the current study are available from the corresponding author on reasonable request.

## Abbreviations

CTBS: Cervical Third of the Buccal Surface
FT: First time evaluation
MTBS: Middle Third of the Buccal Surface
MTLS: Middle Third of the Lingual Surface
NPV: Negative Predictive Value
PPV: Positive Predictive Value
ST: Second time evaluation
